# Milk Replacer Supplementation with Docosahexaenoic Acid from Microalgae Does Not Affect Growth and Immune Status in Goat Kids

**DOI:** 10.3390/ani10071233

**Published:** 2020-07-20

**Authors:** Isabel Moreno-Indias, Lorenzo E. Hernández-Castellano, Davinia Sánchez-Macías, Antonio Morales-delaNuez, Alexandr Torres, Anastasio Argüello, Noemí Castro

**Affiliations:** 1 Animal Production and Biotechnology group, Institute of Animal Health and Food Safety, Universidad de Las Palmas de Gran Canaria, 35413 Arucas, Spain; isabel.moreno@ibima.eu (I.M.-I.); tacho@ulpgc.es (A.A.); noemi.castro@ulpgc.es (N.C.); 2 Unidad de Gestión Clínica de Endocrinología y Nutrición, Instituto de Investigación Biomédica de Málaga (IBIMA), Hospital Universitario Virgen de la Victoria, Universidad de Málaga, 29071 Málaga, Spain; 3 CIBER Fisiopatología de la Obesidad y Nutrición (CIBEROBN), 28029 Madrid, Spain; 4 Department of Animal Science, Aarhus University, AU-Foulum, 8830 Tjele, Denmark; 5 Animal Production and Industrialization Unit, Department of Agroindustrial Engineering, Universidad Nacional de Chimborazo, 060150 Riobamba, Ecuador; dsanchez@unach.edu.ec; 6 Agrobiotechnology Group, Instituto de Productos Naturales y Agrobiología (IPNA), Spanish Research Council (CSIC), 38206 La Laguna, Spain; morales.delanuez@ipna.csic.es; 7 Unit of Animal Production, Pasture, and Forage in Arid and Subtropical Areas. Canary Islands Institute for Agricultural Research, 38200 La Laguna, Spain; atorresk@icia.es

**Keywords:** DHA, omega-3, goat kid, meat quality, chitotriosidase, complement activity

## Abstract

**Simple Summary:**

The consumption of docosahexaenoic acid (DHA) has beneficial effects on human health. Meat from suckling goat kids is highly valuable, especially in Mediterranean countries. Based on this, several strategies have been implemented to increase the content of DHA in foodstuffs such as meat and meat products. Several studies have observed how feeding diverse sources of DHA can improve the fatty acid profile in goat kid meat. However, few studies have focused on the effect of using these DHA supplements on growth and the immune system development in these animals. Consequently, this study aimed to evaluate the effect of different levels of DHA supplementation on growth and the immune system development in newborn goat kids. The current study showed that the DHA supplementation did not affect either growth or the immune status of goat kids during the first 35 days of life.

**Abstract:**

Consumption of polyunsaturated fatty acids (PUFA), especially docosahexaenoic acid (DHA), has beneficial effects for consumers’ health. Consequently, there is an increased interest in enhancing meat fatty acid profiles (i.e., PUFA and DHA content) through diverse nutritional strategies. This study aimed to investigate the effect of supplementing a microalgae-derived product rich in DHA on growth and immune system development in newborn goat kids. In this experiment, newborn goat kids were fed milk replacer (MR) supplemented with three levels of a microalgae-derived product rich in DHA (DHA-Gold^®^, Martek Biosciences, MD, USA). Groups were designed as follows: MR-NS (milk replacer without DHA-Gold^®^ supplementation; *n* = 10), MR-DHA-9 (9 g of DHA-Gold^®^/L milk replacer; *n =* 10) and MR-DHA-18 (18 g of DHA-Gold^®^/L milk replacer; *n =* 10). The immune status of the kids was evaluated by the plasma IgG and IgM concentrations, as well as by the complement system and chitotriosidase activities. Dietary supplementation with DHA did not affect either growth or innate and humoral immunity (*p* > 0.05). This study concludes that supplementation with DHA does not cause negative effects on growth and immune status in newborn goat kids.

## 1. Introduction

Marine microalgae are particularly rich in long-chain (C chain ≥ 20) n-3 polyunsaturated fatty acids (PUFA), especially docosahexaenoic acid (DHA; C22: 6n3) [1]. Consumption of n-3 PUFA, such as DHA, has beneficial effects on human health [2]. Due to the limited consumption of fish and the almost zero utilization of microalgae in western societies, most of the PUFA intake is derived from meat and egg consumption [3]. Consequently, there is greater interest in producing meat and meat products with an optimal ratio between saturated fatty acids, monounsaturated fatty acids and PUFA [4], which would contribute to meeting the dietary recommendations of omega-3 fatty acids [1].

The goat population as well as the meat production from these animals are increasing worldwide [5,6]. Goat meat is considered a high-quality foodstuff [7], especially meat from suckling goat kids, which is highly valued in Mediterranean countries [8]. With the aim of producing meat enriched in n-3 PUFA, different types of fat have been introduced into the diet formulation for goats [9] or goat kids [10]. As showed in these studies, goats and goat kids are able to incorporate dietary n-3 PUFA into different tissues. However, few studies have investigated the effect of supplementing these n-3 PUFA sources on animals’ growth and immune status. 

Goats are born with an immature immune system [11]. In addition, goat placenta (i.e., synepitheliochorial placenta) does not allow the sufficient transfer of immunoglobulins, among other immune components, from the dam to the fetus [12]. Therefore, goat kids are highly susceptible to infectious diseases during the first month of life [13]. Among long chain n-3 PUFA, DHA intake contributes to increasing the levels of diverse DHA-derived metabolites such as protectins (i.e., protectin D, PCTR1, PCTR2 and PCTR3), resolvins (i.e., RvD1, RvD2, RvD3, RvD4, RvD5 and RvD6) and maresins (i.e., marensin 1, marensin 2, MCTR1, MCTR2 and MCTR3) [14], which increases the phagocytic activity of macrophages and promotes the proliferation of diverse lymphocyte T cells (i.e., CD4+ and CD25+ T cells) [14,15]. In addition, DHA intake participates in other physiological processes in mammals [16], contributing to increased newborn vitality and the improvement of cognitive development [17]. DHA-Gold^®^ (Martek Biosciences, MD, USA) is a microalgae-derived product (i.e., *Crypthecodinium cohnii*) rich in DHA (18% w/w minimum), which has been used as a feed ingredient in aquaculture [18], poultry [19,20,21], swine [20] and cattle [22] diets.

Based on these facts, it can be hypothesized that the dietary supplementation with a microalgae-derived product rich in DHA may improve growth and immune system development in newborn goat kids. Therefore, this study aimed to evaluate the effects of feeding milk replacer supplemented with a microalgae-derived product rich in DHA on growth and immune system development in newborn dairy kids during the first 35 days of life.

## 2. Materials and Methods 

### 2.1. Animals, Housing and Treatments

The present study was conducted at the experimental farm of the Veterinary Faculty of the Universidad de Las Palmas de Gran Canaria (Arucas, Spain). The animal experiment procedure was approved by the Ethical Committee of the Universidad de Las Palmas de Gran Canaria (Gran Canaria, Spain) under the project entitled “Reutilización de sueros de quesería; uso ganadero y biotecnológico” (AGL2006-08444/GAN). At birth, 30 Majorera goat kids (15 males and 15 females) were separated from their dams, dried, ear tagged, weighted and the umbilical cord disinfected. After that, kids were bottle-fed using a goat colostrum pool containing 32.59 mg of IgG/mL. The total colostrum amount was equally divided into three meals (i.e., 2, 12 and 24 h after birth) according to the procedure described by Argüello et al. [23]. The total amount of colostrum provided to each goat kid was calculated to provide 4 g IgG/kg of birth body weight (BW), as recommended by Rodríguez et al. [24]. Thereafter, and beginning with the fourth meal (i.e., second day of life), goat kids were distributed according to sex, randomly assigned to one of the three experimental groups, and then received the corresponding experimental diet (Table 1). Kids from the first group (MR-NS) received a commercial milk replacer (MR) formulated for goat kids (Bacilactol Cabritos, Saprogal, La Coruña, Spain; 95.5% dry matter, 23.6% crude protein, and 22.7% ether extract) at 16% (w/w). Animals from the second group (MR-DHA-9) received 9 g of a microalgae-derived product rich in DHA (DHA-Gold^®^, Martek Biosciences, MD, USA; Table 2 shows fatty acid composition) per liter of MR at a final concentration of 16% (w/w: 15.1% MR and 0.9% of DHA-Gold^®^). Finally, animals from the third group (MR-DHA-18) received 18 g of a microalgae-derived product rich in DHA per liter of MR at a final concentration of 16% (w/w; 14.2% of MR and 1.8% of DHA-Gold^®^). Dosages of DHA-Gold^®^ (i.e., 9 and 18 g/L MR) were selected based on a previous study performed by Moreno-Indias et al. [10]. Both experimental diets (i.e., MR-DHA-9 and MR-DHA-18) provided less than 1% of total fiber in the diet, as recommended by Lu and Potchoiba [25]. All animals were fed ad libitum twice daily (0800 and 1700 h) until day 35 of life. Each experimental group was housed in one artificial rearing pen (three artificial rearing pens in total). Each artificial rearing pen was bedded with straw and provided at least 0.3 m^2^ floor space per goat kid. 

### 2.2. Body Weight and Feed Intake Recording and Blood Collection

All goat kids were weighed before blood sample collection. Body weight (BW) was expressed in kilograms (MOBBA, Barcelona, Spain; accuracy, 5 g). Feed intake was recorded for every meal and it was calculated as the amount of either MR-NS, MR-DHA-9 or MR-DHA-18 offered to the animals minus leftovers. Individual feed intake was then calculated by dividing the total feed intake by the number of animals in each experimental group. Individual feed intake was used to calculate the individual microalgae-DHA intake in the three experimental groups. From birth to day 10 of life, blood samples were collected daily from the jugular vein. Afterwards, blood samples were collected every five days until day 35 of life. Blood samples (5 mL) were collected in both heparinized tubes for plasma collection, and glass tubes for serum collection. Immediately after sampling, heparinized tubes were centrifuged (2136× *g*, 15 min, 4 °C) and the resulting plasma was aliquoted and stored at −20 °C. Glass tubes were stored at room temperature (20 °C) for 2 h and then centrifuged (2136× *g*, 15 min, 4 °C). The resulting serum was aliquoted and stored at −20 °C. 

### 2.3. Immune Variables Measured in Plasma and Serum

Plasma immunoglobulin concentrations (IgG and IgM) were measured using goat IgG and IgM ELISA commercial kits (Bethyl Laboratories, Montgomery, TX, USA) and the results were expressed in mg/mL. Serum complement system activity was measured according to Moreno-Indias et al. [26] with modifications [10]. To measure the total complement system activity, DGHB++ buffer (HEPES Gelatin Veronal Buffer, with Ca++ and Mg++: 5 mM HEPES, 71 mM NaCl, 0.15 mM CaCl2, 0.5 mM MgCl2, 2.5% (w/v) glucose, 0.1% (w/v) gelatin, pH 7.4) was used. Similarly, to determine the alternative pathway, DGHB-Mg-EGTA buffer (4.2 mM HEPES, 59 mM NaCl, 7.0 mM MgCl2, 2.08% (w/v) glucose, 0.08% (w/v) gelatin, 10 mM EGTA, pH 7.4) was used. Rabbit red blood cells (RRBC) were diluted at 5% in the corresponding buffer and then added (100 µL) to the serum samples (previously diluted in the correspondent buffer at 5%, 100 µL) in a microtiter plate. The mixture was incubated at 37 °C for 1 h. After this time, cells were removed by centrifugation (2500 g, 5 min), and the absorbance of the supernatant was measured at 405 nm using a microplate reader. Complete hemolysis was achieved by mixing the cells with distilled water (100 µL), and spontaneous lysis was performed by mixing the diluted RRBC with the corresponding buffer. Complement-induced hemolysis of RRBC by the test sera was defined as follows: (A405 sample − A405 spontaneous lysis)/ (A405 100% hemolysis − A405 spontaneous lysis) * 100. Plasma chitotriosidase (ChT) activity was measured according to Argüello et al. [27], using a fluorimeter (Perkin Elmer, Norwalk, CT) at 365 nm for excitation and 450 nm for emission. The ChT activity was expressed as nMol of substrate hydrolysed/mL/h.

### 2.4. Statistical Analysis

All statistical analyses were performed using SAS (version 9.3; SAS Institute Inc., Cary, NC) based on a linear mixed model with repeated measures (PROC MIXED). The model included the supplementation of a microalgae-derived product rich in DHA, days of life and their interaction. Goat kid was set as the repeated subject and sex was set as random effect. Model assumptions were verified based on residual plots and tests for normality. A Tukey–Kramer test was used to evaluate differences between groups. Significance was set as *p* < 0.05. Unless specified, results are presented as least squares means (LS-means) ± standard error of the mean (SEM).

## 3. Results

In this study, the average individual feed intake was 1.26 ± 0.32, 1.30 ± 0.35 and 1.29 ± 0.41 L/day in animals from the MR-NS, MR-DHA-9 and MR-DHA-18 groups, respectively (data expressed as means ± SD). In addition, the average individual microalgae-DHA intake was 2.29 ± 0.28 and 4.62 ± 0.46 g/day in animals from the MR-DHA-9 and MR-DHA-18 groups, respectively (data expressed as means ± SD).

### 3.1. Supplementation of a Microalgae-Derived Product Rich in DHA Did Not Affect Growth or Feed Intake in Goat Kids.

**Figure 1:**
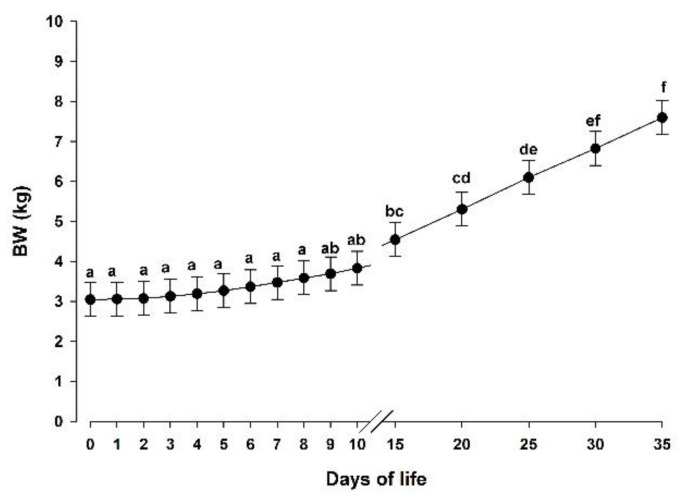
Body weight (BW) in the three groups together (MR-NS, MR-DHA-9 and MR-DHA-18; *n =* 30) from day 0 to day 35 of life. Different lowercase letters (a–f) indicate significant (*p* < 0.05) differences between time points. Results are presented as least squares means ± standard error of the mean. *p*-values are shown in Table 3. MR-NS: milk replacer without supplementation of a microalgae-derived product rich in DHA; MR-DHA-9: milk replacer with supplementation of 9 g of a microalgae-derived product rich in DHA; MR-DHA-18: milk replacer with supplementation of 18 g of a microalgae-derived product rich in DHA.

At birth, no differences in BW were detected between groups (3.25 ± 0.25, 3.00 ± 0.28 and 2.88 ± 0.29 kg in MR-NS, MR-DHA-9 and MR-DHA-18 groups, respectively; *p >* 0.05; data are expressed as LS-means ± SD). In addition, the supplementation of a microalgae-derived product rich in DHA (DHA-Gold^®^) did not affect the final BW at day 35 of life (7.27 ± 0.28, 7.45 ± 0.33 and 7.09 ± 0.35 kg in MR-NS, MR-DHA-9 and MR-DHA-18, respectively; *p >* 0.05; data are expressed as LS-means ± SD). As showed in Table 3, the supplementation of a microalgae-derived product rich in DHA (DHA-Gold^®^) did not affect BW during the first 35 days of life (*p* > 0.05). As showed in Figure 1, BW increased during the entire experimental period (*p* < 0.05). 

### 3.2. Supplementation of a Microalgae-Derived Product Rich in DHA Had No Effect on Blood Immunoglobulin Concentrations in Goat Kids

**Figure 2:**
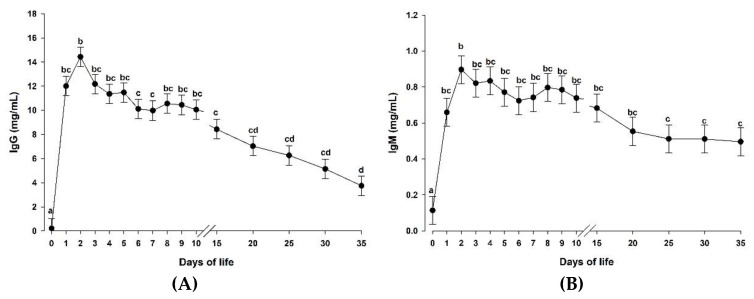
Plasma immunoglobulin G (IgG) concentration (**A**) and immunoglobulin M (IgM) concentrations (**B**) in the three groups together (MR-NS, MR-DHA-9 and MR-DHA-18; *n =* 30) from day 0 to day 35 of life. Different lowercase letters (a-d) indicate significant (*p* < 0.05) differences between time points. Results are presented as least squares means ± standard error of the mean. *p*-values are shown in Table 3. MR-NS: milk replacer without supplementation of a microalgae-derived product rich in DHA; MR-DHA-9: milk replacer with supplementation of 9 g of a microalgae-derived product rich in DHA; MR-DHA-18: milk replacer with supplementation of 18 g of a microalgae-derived product rich in DHA.

Table 3 shows the plasma IgG concentrations in the three studied groups during the entire experimental period. The supplementation of a microalgae-derived product rich in DHA (DHA-Gold^®^) did not affect plasma IgG concentrations in the experimental groups (*p* > 0.05). As showed in Figure 2A, plasma IgG concentrations increased rapidly after colostrum feeding (*p* < 0.05) and decreased progressively until the end of the experimental period (day 35).

As described above for the IgG concentrations, the supplementation of a microalgae-derived product rich in DHA (DHA-Gold^®^) had no effect on the IgM concentrations (Table 3, *p* > 0.05). Similarly to IgG concentrations, IgM concentrations were clearly influenced by time (*p* < 0.05; Figure 2B). The highest IgM concentrations were obtained at day 2 (*p* < 0.05) and then they remained constant from day 3 until the end of the experimental period (day 35).

### 3.3. Neither the Complement System nor the Chitotriosidase Activities Were Affected in Goat Kids Supplemented with a Microalgae-Derived Product Rich in DHA

**Figure 3:**
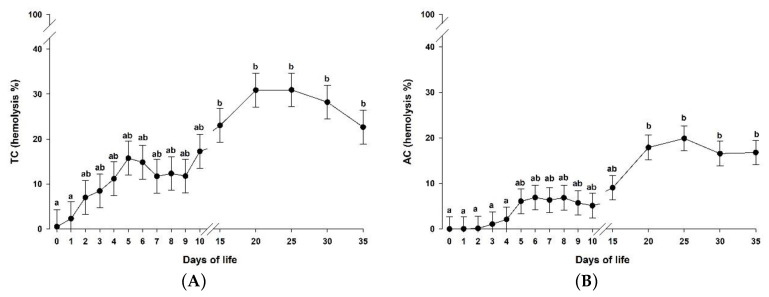
Serum total complement system (TC) activity (**A**) and alternative pathway (AC) activity (**B**) in the three groups together (MR-NS, MR-DHA-9 and MR-DHA-18; *n =* 30) from day 0 to day 35 of life. Different lowercase letters (a-b) indicate significant (*p* < 0.05) differences between time points. Results are presented as least squares means ± standard error of the mean. *p*-values are shown in Table 3. MR-NS: milk replacer without supplementation of a microalgae-derived product rich in DHA; MR-DHA-9: milk replacer with supplementation of 9 g of a microalgae-derived product rich in DHA; MR-DHA-18: milk replacer with supplementation of 18 g of a microalgae-derived product rich in DHA.

The supplementation of a microalgae-derived product rich in DHA (DHA-Gold^®^) did not affect either the total complement system activity or the activity of the alternative pathway (Table 3; *p* > 0.05). However, both of them were affected by time in the studied groups (Figure 3A and 3B, respectively; *p* < 0.05). As can be observed, the complement system activity was not detectable at birth, increasing the activity of both the total complement system as well as the activity of the alternative pathway until day 15 and 20, respectively. Then, both activities remained constant until the end of the experimental period.

Chitotriosidase activity (Table 3) was not affected either by the supplementation of a microalgae-derived product rich in DHA (*p* > 0.05) or by time (*p* > 0.05).

## 4. Discussion

The first month of life is considered one of the most challenging periods for ruminants [28]. During this period, milk represents the main energy source for these animals [11,29]. Nowadays, there is an increasing number of high production dairy farms, in which artificial rearing is chosen as a way to increase the amount of milk available for processing [30,31] and simplify animal management [32]. Consequently, it is important to supplement balanced and optimal milk replacers in order to avoid negative consequences on animal health and performance [33]. Docosahexaenoic acid is widely recognized for its central role in the development of cognitive functions as well as in the development of the immune system in humans and animals [10,34]. In fact, once DHA is incorporated into phospholipids, it can be metabolized by neutrophils into prostaglandins, leukotrienes, thromboxanes, maresins, protectins, and resolvins [35,36]. Docosahexaenoic acid as well as their metabolites promotes neutrophil migration and phagocytic capacity, as well as the production of reactive oxygen species and cytokines [14]. In addition, DHA affects macrophages by increasing the production and secretion of cytokines and chemokines, the capacity of phagocytosis, and the polarization into classically activated or alternatively activated macrophages [14]. Based on the beneficial effects of DHA on cognitive functions and the immune system, it was expected that animals supplemented with microalgae-DHA would have higher BW than those not supplemented with that product. However, the dietary supplementation with a microalgae-derived product rich in DHA used in this study did not affect the BW of the goat kids during the first 35 days of life. These findings are in agreement with previous studies performed by our group [10], although in that study the authors used a lower DHA supplementation. Voluntary feed intake of milk replacer by pre-ruminant animals is highly influenced by the dry matter content of the milk replacer [37]. The present study provided a balanced diet based on dry matter, which could explain the lack of differences in BW. As described by Lu and Potchoiba [25], fiber content in MR for goat kids higher than 1% causes diarrhea and therefore is not recommended. Therefore, it is unlikely that a higher dose of DHA-Gold^®^ (i.e., 27 g/L MR) will improve BW in goat kids, as the fiber content of the MR will be also increased above 1%. 

In addition to the role of DHA as a source of energy, this fatty acid contributes also in several immune processes [14,38]. In the current study, the dietary supplementation with a microalgae-derived product rich in DHA did not affect any of the measured immune variables in goat kids. Similar results were obtained in previous studies performed in lambs supplemented with either fish oil or safflower oil [39]. However, a similar study performed in piglets showed increased IgG and IgM concentrations as a consequence of the dietary supplementation of microalgae extracts and fish oils [40]. As has been previously described, immunoglobulins are produced by B lymphocytes, being one of the major components of the adaptive immune system. Ruminants are considered either agammaglobulinemic (cows) or hypogammaglobulinemic (goats and sheep) at birth, and therefore they strongly depend on the immunoglobulins provided by the dam via colostrum [28,41,42]. In the present study, colostrum feeding during the first 24 h of life increased the IgG and IgM concentrations in the three studied groups, reaching the highest concentrations on day 2 of life. These levels were similar to those described by Moreno-Indias et al. [10] and Rodríguez et al. [24] in the same breed and those described by Mellado et al. [43] and Batmaz et al. [44] in Granadina and Saanen breeds, respectively. However, this passively transferred protection only lasts for a few weeks, where ruminants must be able to have an adequate immune system development in order to synthesize their own defenses. In piglets, B lymphocytes are the first lymphocytes detected in the bloodstream and they can be found in the thymus at birth [45]. However, the goat kid immune system starts to slowly produce B lymphocytes, and consequently immunoglobulins, on the third week of life [46]. This fact may explain the lack of effect of the DHA supplementation on an immune system in development and not fully active. Based on these facts, it seems that the effect of DHA supplementation on the immune system development depends on several factors, such as species and age of the animals, among others [47]. In addition, it also seems that the immune system development in goat kids is not affected by the level of DHA-Gold^®^ supplementation (9 g/L MR vs. 18 g/L MR).

Other immune components, such as the complement system activity and the ChT activity, play a fundamental role in the innate immune response, acting as a part of the host defense in newborn ruminants [48,49]. Despite that fact that the complement system activity was not affected by the addition of either 9 or 18 g of DHA-Gold^®^/L MR, activities of both the total complement system as well as the alternative pathway were detectable after the first days of life. These results are different from those reported by Castro et al. [50], who did not detect any complement system activity in goat kids fed with milk replacer during the first 60 days of life. Nevertheless, these authors described that the addition of conjugated linoleic acid into milk replacer caused an earlier activation of the complement system. Differences between the results showed by Castro et al. [50] and the ones described in the present study might be attributed to the different technique used to determine the complement system activity. Chitotriosidase is an important component of innate immunity against chitin-containing pathogens [27], being able to cleave chitin present in the cell walls of fungi and nematodes [51]. Consequently, ChT activity has been strongly related to the host defense [52,53]. In fact, this enzyme has been investigated in several species, such as humans [54], goats [27,55,56] and sheep [29,48]. In the present study, the milk replacer supplementation with DHA-Gold^®^ did not affect the ChT activity, even when 18 g of DHA-Gold^®^/L MR was used (w/w: 14.2% of milk replacer and 1.8% of DHA-Gold^®^). The values reported in this study are in agreement with those reported by Moreno-Indias et al. [10] in goat kids, which showed constant ChT activity during the first 35 days of life.

Based on the results described above, the dietary supplementation with a microalgae-derived product rich in DHA did not cause any effect on the humoral immune response in newborn goat kids, even when 18 g of DHA-Gold^®^/L MR was used (w/w; 14.2% of milk replacer, and 1.8% of DHA-Gold^®^). Thus, DHA supplementation in humans during pregnancy and lactation has been described to not affect infants’ humoral immune response [57], although changes in the lymphocyte subset profile and cytokine production were observed in that study. Similarly, the dietary supplementation with DHA has been described to improve immune function in pigs [58], increasing the number of lymphocytes and monocytes. As ChT activity can be used as a marker for macrophage activation [59], it seems that the supplementation of DHA did not cause either an earlier or higher activation of macrophages in this study. Therefore, it can be speculated that DHA supplementation affects differently the activation of macrophages in pigs than in goats.

## 5. Conclusions

This study concludes that the supplementation of DHA from microalgae does not affect either growth or immune status in goat kids during the first 35 days of life. This study also concludes that the use of DHA from microalgae as a dietary supplement to improve either growth or the immune status in goat kids is not clear and should be further investigated in future studies.

## Figures and Tables

**Table 1:** Chemical composition of the experimental diets (g/L).

	MR-NS	MR-DHA-9	MR-DHA-18
Crude protein	3.95	3.84	3.72
Fat	3.80	4.04	4.28
Total fiber	0.02	0.39	0.77
Ash	1.37	1.36	1.35

MR-NS: milk replacer without supplementation of a microalgae-derived product rich in docosahexaenoic acid (DHA); MR-DHA-9: milk replacer with supplementation of 9 g of a microalgae-derived product rich in DHA; MR-DHA-18: milk replacer with supplementation of 18 g of a microalgae-derived product rich in DHA.

**Table 2:** Product specifications and fatty acid profile of DHA-Gold^®^ (DHA-Gold^®^, Martek Biosciences, MD, USA), showing only fatty acids greater than 1% of the total fatty acids.

**Product specifications ^1^**	
DHA (C22:6n3) content	min. 18%
Total fat	min. 35%
Unsaponificable matter	max. 8%
Insoluble impurities	max. 5%
Free fatty acids	max. 5%
Moisture	max. 6%
**Fatty acid profile**	**g/100g of DHA-Gold^®^**
C14:0	5.90
C16:0	13.1
C22:5n6	6.84
C22:6n3	19.4

^1^ Adapted from DHA-Gold^®^, Martek Biosciences, MD, USA.

**Table 3:** Least squares means of body weight (BW), immunoglobulin G (IgG) and M (IgM) concentrations, chitotriosidase (ChT) activity as well as total complement (TC) system and alternative pathway (AC) activities in the MR-NS (*n =* 10), MR-DHA-9 (*n =* 10) and MR-DHA-18 (*n =* 10) groups.

Variable	MR-NS	MR-DHA-9	MR-DHA-18	SEM	Fixed Effects (*p*-Value)		
					TRT	D	TRT × D
BW (kg)	4.27	4.17	4.22	0.38	0.926	<0.001	0.255
IgG (mg/mL)	9.58	8.73	8.21	0.51	0.176	<0.001	0.165
IgM (mg/mL)	0.73	0.66	0.57	0.05	0.069	<0.001	0.937
TC (hemolysis%)	18.7	15.3	14.6	2.30	0.130	<0.001	0.984
AC (hemolysis%)	9.71	8.74	7.94	2.51	0.502	<0.001	0.420
ChT (nmol/mL/h)	1619	1605	1647	108	0.961	0.13	0.976

MR-NS: milk replacer without supplementation of a microalgae-derived product rich in DHA; MR-DHA-9: milk replacer with supplementation of 9 g of a microalgae-derived product rich in DHA; MR-DHA-18: milk replacer with supplementation of 18 g of a microalgae-derived product rich in DHA; SEM= Standard error of the mean; TRT = supplementation of a microalgae-derived product rich in DHA; D = days of life; TRT×D = interaction between TRT and D.
